# Dietary Selenium and Its Antioxidant Properties Related to Growth, Lipid and Energy Metabolism

**DOI:** 10.3390/antiox11071402

**Published:** 2022-07-19

**Authors:** María Luisa Ojeda, Fátima Nogales

**Affiliations:** Department of Physiology, Faculty of Pharmacy, Seville University, 41012 Seville, Spain; fnogales@us.es

Selenium (Se) is an essential trace element mainly known for its antioxidant, anti-inflammatory and anti-apoptotic properties. Se plays its biological role by forming part of 25 selenoproteins; most of them are known for their antioxidant properties, such as glutathione peroxidases (GPxs), thioredoxin reductases (TXNRDs) and selenoprotein P (SelP). This antioxidant activity is linked to endocrine regulation, lipid and glucose homeostasis, appetite, growth and energy balance. Moreover, some of them intricately regulate the functioning of the endocrine system and intracellular signaling. Eight selenoproteins, among them deiodinase 1 (Dio1), Dio2, and Dio3, influence the synthesis of thyroid hormones affecting the basal metabolic rate. GPxs and TXNRDs are essential for correct β-cell function and insulin secretion; furthermore, Se regulates the transcription levels of the hypothalamic and GH/IGF axis-related genes responsible for growth promotion [1]. Apart from these endocrine actions, which affect lipid, protein and glucose homeostasis, Se modulates adipogenesis via GPx1 SREBP1, SelP and SelW, since this is a process where reactive oxygen species (ROS) are used as transmitters [2,3]. These effects are even more important during intense growth periods, such as gestation, lactation, adolescent and embryogenic periods, where tissues are growing and in some cases developing [1,2].

The health and biological effects of Se are well-known for being dependent not only on its chemical forms, which can be inorganic (e.g., selenite) and organic (e.g, selenomethionine) species [4]. However, they are also dependent on dosage since a high dietary Se intake by dams induces insulin resistance (IR) and adipose tissue depots in the offspring, whereas low-Se diets lead to metabolic disorders related to type 1 diabetes and catabolic processes [1]. Recently, selenium nanoparticles (SeNPs) have been reported to have many unique biological activities. SeNPs have a wide surface area that is possibly responsible for their functionality and permeability, being more biologically available with fewer amounts of Se, making them less toxic and highly effective [4].

This Special Issue, entitled “Dietary Selenium and Its Antioxidant Properties Related to Growth, Lipid and Energy Metabolism” (ISSN 2076-3921), which belongs to the section “Health Outcomes of Antioxidants and Oxidative Stress”, presents papers concerning Se and selenoproteins, and their relationship with oxidative stress (OS), growth and/or lipid and energy metabolism, which could lead to metabolic disorders. It includes five research papers and four reviews from distinguished experts in this area. These contributions address different aspects of Se on adipose tissue as well as obesity and adipose-tissue insulin sensitivity in adults and adolescent animals, which could affect their reproductive function. Since this element is crucial for correct reproductive physiology, its relationship to mastitis, offspring health and metabolic syndrome (MetS) fetal programing are also described. Other studies analyzed the effects of Se supplementation in fatty liver-associated disorders. In this context, two important clinical trials used urinary and dietary Se to analyze non-alcoholic fatty liver disease (NAFLD) progression and dyslipidemia. Finally, two studies analyzed the use of SeNPs, pointing to their important role in immunomodulation and as a metabolic regulator in aquaculture.

The first article is entitled “Selenium Nanoparticles (SeNPs) Immunomodulation Is More Than Redox Improvement: Serum Proteomics and Transcriptomic Analyses” [5]. This article, using zebrafish, studies the molecular mechanisms involved in SeNP-induced immunomodulation in healthy or disease conditions. The authors concluded that the immunomodulatory effects of SeNPs are highly related not only to antioxidant activity, but also to lipid metabolism modulation. Interestingly, the biological functions enhanced by SeNPs are almost identical in healthy and disease conditions. However, while the SeNPs suppress ROS in healthy individuals, they promote ROS formation during disease condition in order to increase the defense against pathogens. This fact indicates a close relationship between immune and redox functions, where SOD and NFkβ pathways are implicated.

The second article, entitled “Selenium Supplementation during Puberty and Young Adulthood Mitigates Obesity-Induced Metabolic, Cellular and Epigenetic Alterations in Male Rat Physiology” [2], evaluates the efficacy of Se supplementation, specifically during puberty, against the obesity-induced deregulation of metabolic, cellular and epigenetic parameters in epididymal fat and/or sperm cells in a rat model. By using a high-fat diet in male rats during puberty, the authors reported a high adipocyte size, OS, the deregulated expression of genes associated with inflammation (Adiponectin, IL-6, TNF-α), adipogenesis (CEBPα), estrogen biosynthesis (CYP19) and epigenetic processes in epididymal adipose tissue, and altered microRNA expression vital for spermatogenesis in sperm cells (miR-15b and miR-497). Importantly, Se supplementation significantly decreased OS and mitigated these molecular/epigenetic alterations in epididymal adipose tissue or sperm cells. It suggests that Se supplementation during puberty could improve male physiology in the context of obesity.

The third article, entitled “Obesity Hinders the Protective Effect of Selenite Supplementation on Insulin Signaling” [3], is a complex, multidisciplinary and interesting study that analyzed the use of Selenite supplementation in mice to avoid the effects of high-fat diets associated with obesity, OS, and IR. The authors also studied this effect in cultivated 3T3-L1 pre-adipocytes. Moreover, the authors evaluated GPx3 mRNA expression and its relationship to adiposity in human subcutaneous adipose tissue samples and investigated whether selenite supplementation was able to counteract lipotoxicity-induced IR in vitro and obesity in vivo. They confirmed that GPx3 mRNA expression in adipose tissue correlates with BMI in humans. They found that, in cultivated 3T3-L1 pre-adipocytes, Se treatment attenuates IR with enhanced GPx3 and IRS expression and adipocyte differentiation and that, when feeding obese mice, selenite supplementation improved adipocyte morphology but it did not alter adipose tissue insulin sensitivity despite they exhibited increased insulin content in the pancreas. Overall, while selenite protects against IR in vitro, the effect of selenite supplementation in vivo is not exactly the same.

The fourth article, entitled “Association of Urinary and Dietary Selenium and of Serum Selenium Species with Serum Alanine Aminotransferase in a Healthy Italian Population” [6], used a cross-sectional study with 137 healthy blood donors living in Northern Italy to analyze the relationship of selenium, in its different administration forms, with serum alanine aminotransferase (ALT) levels, a marker of NAFLD. The authors found that urinary Se levels and dietary Se intake were positively correlated with ALT, while total serum Se was inversely associated with ALT up to 120 µg/L. Concerning the different serum Se species, ALT positively correlated with two organic forms, selenocysteine and GPx-bound Se, which showed a U-shaped relation with the inorganic tetravalent form, selenite, and an inverse association with human serum albumin-bound Se. These results suggest that overall exposure to Se, and more specifically to some of its chemical forms, is positively associated with ALT, even at safe levels, suggesting that the low-dose Se overexposure is associated with NAFLD.

The fifth article, entitled “Associations between Urinary and Dietary Selenium and Blood Metabolic Parameters in a Healthy Northern Italy Population” [7], evaluates Se status with different health endpoints by quantifying urinary Se excretion and dietary Se intake in 137 healthy, non-smoking blood donors living in the Northern Italian province of Reggio Emilia. They found that dietary and urinary Se levels were correlated, although the association of the two indicators with health endpoints tended to diverge. Thus, there is a positive association between urinary Se and blood triglyceride, LDL-cholesterol, and glucose levels and an negative association with HDL-cholesterol. Concerning dietary Se, there is only a slightly positive association with glycemic levels. Based on this, it could be suggested that higher Se exposure, even at levels not exceeding the tolerable upper intake, is adversely associated with the blood glucose levels and the lipid profile.

The sixth article is a review entitled, “The Role and Mechanisms of Selenium Supplementation on Fatty Liver-Associated Disorder” [8]. This narrative review studies the role of Se in the regulation of the different stages of NAFLD, the most frequent chronic liver disease that still does not have an effective treatment. This review also summarizes the most relevant clinical trials in order to highlight the potential roles of Se in NAFLD treatment, and thus clarifies the correlation between Se and NAFLD.

The seventh article, entitled “Role of Selenium and Vitamins E and B9 in the Alleviation of Bovine Mastitis during the Periparturient Period” [9], is a review that studies mastitis, an inflammation of the mammary gland, which commonly occurs in dairy cattle during the periparturient period. In this period, dairy cattle experience physiological and endocrine changes, severe negative energy balance and OS. In addition, to maintain a successful lactation and to combat this negative energy balance, excessive fat mobilization occurs. Among other mechanisms, OS impairs the immunity and anti-inflammatory efficiency of periparturient dairy cattle, increasing their susceptibility to the development of mastitis. In this narrative review, the authors discuss the development of bovine mastitis and its major causes with a special emphasis on OS, as well as the antioxidant, immunomodulatory and anti-inflammatory properties of Se and vitamins E and B9 and their role in the control of bovine mastitis.

The eighth article is entitled “The Role of Selenoprotein Tissue Homeostasis in MetS Programming: Energy Balance and Cardiometabolic Implications” [1]. It describes the main selenoproteins related to IR and MetS generation, which modulate not only ROS levels and the energetic sensor AMP-activated protein kinase (AMPK), but also the nuclear transcription factor kappa-B (NF-kB), leading to changes in inflammation production. This review introduces the selenoproteins implicated in the correct synthesis of insulin, thyroid hormones and IGF-1 and those related to the endocrine signals that regulate appetite and energy balance. Secondly, this narrative review provides an overview of existing evidence, based mainly on experimental research, that strengthens the fact that maternal MetS leads to changes in Se tissue deposits and antioxidant selenoproteins’ expression in their offspring. It also explores how these changes lead to energetic and metabolic alterations that, according to the metabolic programming theory, will produce cardiovascular and metabolic diseases later in life, mainly affecting heart development. In this context, MetS offspring presents a profile similar to that of diabetes type 1, which also appeared when dams were exposed to low-Se dietary supply; therefore, dietary maternal Se supplementation should be taken into account if, during gestation and/or lactation periods, there are suspicions of endocrine energy imbalance in the offspring, such as MetS. However, more studies are still necessary.

The ninth article, entitled “Selenium Nanoparticles as a Natural Antioxidant and Metabolic Regulator in Aquaculture: A Review” [4], is a comprehensive review that describes the recent progress in the use of SeNPs in aquaculture. It explains the low margin between the benefits and the toxicity of Se, and how SeNPs are included in aquafeed in a dose-specific manner since novel SeNPs are characterized by their low toxicity and high functionality. In this article, the multiple roles of SeNPs are presented and discussed with a particular focus on (1) the growth-promoting and feed utilization effects, (2) the metabolic regulation roles, (3) the antioxidative and physiological aspects, and (4) the Se nanoparticles role as antistress agents.

This Special Issue collected articles and reviews that reveal the importance of Se and selenoproteins not only in oxidative and inflammatory balance, but also in endocrine regulation, lipid and glucose homeostasis, appetite, reproduction, and growth and energy balance; these effects are even more important during intense growth and developmental periods. Se supplementation has been suggested as a possible therapy for different metabolic health problems. Moreover, Se urinary and dietary levels measurement are proposed for use as clinical markers of NAFLD evolution and dyslipidemias. Important new and growing evidence has been compiled regarding their relationship to Se adipogenesis and obesity, indicating that higher selenite concentrations are needed to profoundly improve metabolism in established obesity. Finally, the potential benefits of using SeNPs to improve Se bioavailability and decrease toxicity were demonstrated, especially in the aquaculture industry.
